# Effect of Altered Sleep Duration on Sleep Bruxism

**DOI:** 10.7759/cureus.95207

**Published:** 2025-10-23

**Authors:** Yuichiro Yamakawa, Takashi Iida, Yoshihiro Iwata, Masatoshi Iwasaki, Osamu Komiyama

**Affiliations:** 1 Department of Prosthodontics and Oral Rehabilitation, Nihon University School of Dentistry at Matsudo, Chiba, JPN

**Keywords:** electromyography, portable electromyography device, sleep bruxism, sleep deprivation, sleep quality

## Abstract

Introduction: Inconsistent findings have been reported regarding the relationship between sleep bruxism (SB) and sleep quality; therefore, the aim of the present study was to investigate the effect of sleep deprivation (SD) on masticatory muscle activity during sleep.

Methods: Twenty-eight healthy participants were subjected to a five-day experimental protocol involving different sleep conditions, including baseline sleep (BS), one night of SD, and two nights of recovery sleep (RS1 and RS2). A portable electromyography device was used to measure SB events, and a Sleep Profiler device (Advanced Brain Monitoring Inc, Carlsbad, CA, USA) simultaneously recorded sleep parameters. The total sleep time, sleep efficiency, proportion of time spent in each sleep stage, microarousal index, and number of SB events were compared between the BS, RS1, and RS2 periods. The Epworth Sleepiness Scale, Stress Numerical Rating Scale-11, and World Health Organization-Five Well-Being Index for stress were used for subjective assessments, and values were compared between three time points (after BS, SD, and RS1).

Results: The SB event frequency was significantly lower during RS1 than during BS (P < 0.001) and RS2 (P = 0.022). During RS1, the total sleep time (P = 0.039), sleep efficiency (P = 0.047), and proportion of sleep time in N3 (P = 0.019) were significantly higher, whereas the proportion of time spent in N2 was lower (P = 0.032). After SD, sleepiness and stress significantly increased, whereas well-being decreased (P < 0.001).

Conclusion: SD affects SB events, the frequency of which can be reduced by improving sleep quality to lower stress levels.

## Introduction

Sleep bruxism (SB) is defined as rhythmic (phasic) or non-rhythmic (tonic) masticatory muscle activity occurring during sleep and is not considered to be either a movement or sleep disorder in otherwise healthy individuals [1]. Etiological studies have identified caffeine intake, heavy alcohol consumption, anxiety, sleep apnea, psychological stress, and gastro-esophageal reflux as major risk factors associated with SB and have suggested that it is mediated through central rather than peripheral nervous system activity [2]. However, the pathophysiological mechanism underlying SB has yet to be completely elucidated. Furthermore, while some observational studies have used a polysomnography (PSG) system to investigate masticatory activity during sleep [3,4], the findings have not translated to improve the clinical management of SB, and there is currently no established international consensus to guide treatment planning among dental clinicians. Therefore, objectively evaluating SB is necessary in order to establish novel management strategies and clarify the underlying mechanism.

Some studies have investigated correlations between sleep quality and the occurrence of SB; however, the findings have been inconsistent. For example, Ohlmann et al. [5] reported no significant correlation between SB definitively diagnosed using a portable electromyography (EMG)-electrocardiography device and either self-reported chronic stress or sleep quality. Conversely, Câmara-Souza et al. [6] suggested that SB may be associated with low oral health-related quality of life and poor sleep quality. Those observational studies only measured sleep quality subjectively; therefore, it is essential to investigate the effect of sleep quality on SB objectively through interventional research.

Sleep quality has been shown to be influenced by various factors, including genetics, alcohol consumption, smoking, and stress [7]. In addition, poor sleep quality has been suggested to be a risk factor associated with various sleep disorders [8].

To alter sleep quality, some studies have incorporated sleep deprivation (SD) into their experimental design and demonstrated that SD affects pain perception and stress responses [9,10]. More specifically, in the field of dentistry, sleep restriction has been shown to influence somatosensory sensitivity in the orofacial area [11,12].

The aim of this study was to investigate the effect of SD on masticatory muscle activity during sleep, as the findings could lead to the establishment of a novel strategy for the management of SB based on the modification of sleep schedules as a lifestyle intervention and could help elucidate the underlying mechanisms. The hypothesis was that SD would alter masticatory muscle activity during sleep.

## Materials and methods

Participants

This study included 28 healthy individuals (15 men and 13 women; mean ± SD age: 30 ± 3 years). The exclusion criteria were as follows: medical or psychological problems (including psychiatric diseases, epilepsy, cardiovascular disease, or prior SD), pregnancy, current smoking, medication use (analgesics, antidepressants, or hypnotics) within 48 hours before the investigation, and excessive caffeine or alcohol consumption. The required sample size of 28 was calculated using G*Power 3.1 (Heinrich-Heine-Uni-versität Düsseldorf, Germany) based on an alpha value (α, probability of making a type I error) and power (1-β, the probability of not making a type II error) of 0.05 and 0.80, respectively, with a medium effect size of 0.25 according to the criteria described by Cohen [13].

Informed consent was obtained from all participants before the experiment. The study protocol was approved by the Ethics Committee of Nihon University School of Dentistry at Matsudo (approval number: EC 20-031; date of approval: December 10, 2020) and was conducted in accordance with the tenets of the Declaration of Helsinki and its later amendments.

Experimental design

**Figure 1:**
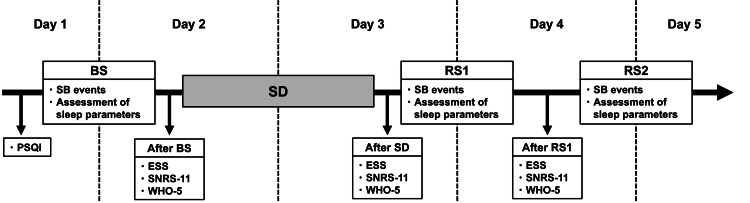
Diagram of the study’s design. Assessment of sleep parameters included the microarousal index, proportion of time spent in each sleep stage, and sleep efficiency. After BS: after baseline sleep; after RS1: after recovery sleep 1; after SD: after sleep deprivation; BS: baseline sleep; ESS: Epworth Sleepiness Scale; SNRS-11: Stress Numerical Rating Scale-11; PSQI: Pittsburgh Sleep Quality Index; RS1: recovery sleep 1; RS2: recovery sleep 2; SD: sleep deprivation; WHO-5: World Health Organization-Five Well-Being Index.

Over a period of five days, the participants were subjected to different sleep conditions, and measurements were conducted at various time points (Figure 1). All participants underwent unattended, at-home sleep recordings throughout the experimental period. From the night of Day 1 to the morning of Day 2, the participants were instructed to sleep as they normally would; this period was defined as baseline sleep (BS). From the night of Day 2 to the morning of Day 3, the participants underwent voluntary SD, during which they were instructed to remain awake throughout the night. Following the night of SD, participants slept as usual from the night of Day 3 to the morning of Day 4 (recovery sleep 1 (RS1)) and from the night of Day 4 to the morning of Day 5 (recovery sleep 2 (RS2)).

Measurements

The Pittsburgh Sleep Quality Index (PSQI) [14] was used to assess sleep quality before BS. The Epworth Sleepiness Scale (ESS) [15], Stress Numerical Rating Scale-11 (SNRS-11) [16], and World Health Organization-Five Well-Being Index (WHO-5) [17,18] were used to subjectively assess sleepiness, stress, and mental health, respectively, at various points, including after BS, after SD, and after RS1.

While sleeping each night, the participants were instructed to simultaneously wear a single-channel portable EMG device (Sunstar BUTLER GrindCare, Sunstar Suisse SA, Etoy, Switzerland) and a sleep assessment device (Sleep Profiler™, Advanced Brain Monitoring Inc, Carlsbad, CA, USA). The single-channel portable EMG device monitored temporalis muscle activity during clenching or grinding to calculate the number of SB events per hour. The EMG signal was bandpass-filtered after sampling at a frequency of 2 kHz. Baseline EMG amplitudes were calculated from the 80 filtered signal points immediately before a preset point. An SB event was recorded each time the EMG amplitude exceeded three times the standard deviation calculated from the baseline EMG amplitudes. SB events per hour were counted by the portable EMG device each night and digitally recorded.

The total sleep time, sleep efficiency, proportion of time spent in each sleep stage (rapid eye movement (REM) and N1, N2, and N3 non-REM stages), and microarousal index were measured using a battery-powered recorder designed to acquire three frontopolar electroencephalography (EEG) signals between AF7-AF8, AF7-Fpz, and AF8-Fpz. The EEG signals were sampled at 256 Hz with a gain of ±1,000 μV and filtered using a 0.1 Hz high-pass and 80 Hz low-pass filter. For auto-staging, machine learning algorithms were used to establish the relationships between the EEG power extracted from the delta (1-3.5 Hz), theta (4-6.5 Hz), alpha (8-12 Hz), sigma (12-16 Hz), beta (18-28 Hz), and EMG (40-128 Hz with a 67 Hz low-pass hardware filter) bands for the staging of each 30 s epoch. The auto-staging methodology has been validated in previous studies based on comparisons with PSG visual scoring data [19,20].

Statistical analysis

All data are presented as the mean and standard deviation. For the ESS, SNRS-11, and WHO-5 scores, comparisons were made between time points (after BS, after SD, and after RS1). For the total sleep time, sleep efficiency, proportion of time spent in each sleep stage, microarousal index, and SB event frequency, comparisons were made between the BS, RS1, and RS2 periods. The Shapiro-Wilk test was used to assess normality. Variables that met the assumption of normality were analyzed using one-way repeated measures analysis of variance, whereas non-normally distributed variables were analyzed using the Friedman test. The Bonferroni method was employed for multiple comparisons. P < 0.05 was considered statistically significant. All analyses were performed using the SigmaPlot 14.5 package (Systat Software, San Jose, CA, USA).

## Results

**Figure 2:**
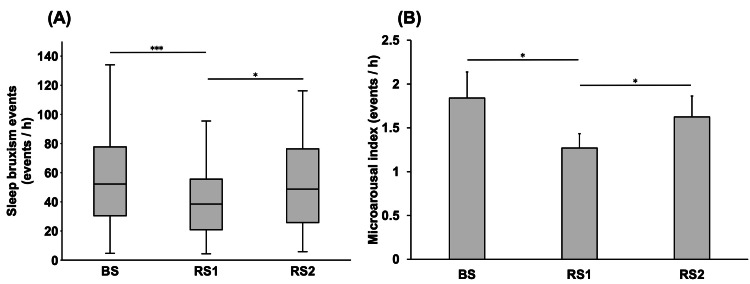
Comparison of the frequency of SB events (A) and the microarousal index (B) on each day. BS: baseline sleep; RS1: recovery sleep 1; RS2: recovery sleep 2; SB: sleep bruxism. *P < 0.05; ***P < 0.001.

The mean PSQI score of all participants before BS was 2.9 ± 0.24. Figure 2A shows the comparison of the frequency of SB events per hour between the BS, RS1, and RS2 periods. The frequency of SB events was significantly lower during RS1 (38.7 ± 5.81) than during BS (54.7 ± 8.10) and RS2 (51.9 ± 7.05) (P < 0.001 and P = 0.022, respectively).

Figure 2B shows the comparison of the microarousal index between the BS, RS1, and RS2 periods. The microarousal index was significantly lower during RS1 (1.27 ± 0.16) than during BS (1.84 ± 0.29) and RS2 (1.62 ± 0.23) (P = 0.021 and P = 0.032, respectively).

**Figure 3:**
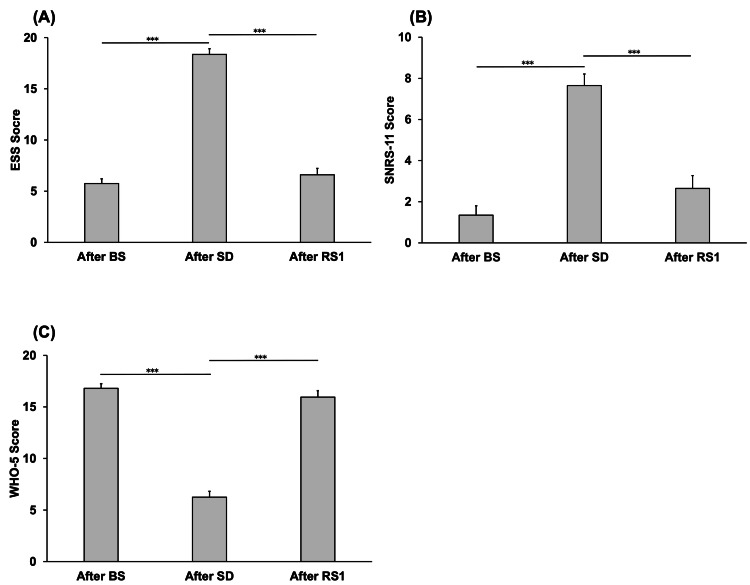
Comparison of the ESS (A), SNRS-11 (B), and WHO-5 (C) scores on each day. After BS: after baseline sleep; after RS1: after recovery sleep 1; after SD: after sleep deprivation; ESS: Epworth Sleepiness Scale; SNRS-11: Stress Numerical Rating Scale-11; WHO-5: World Health Organization-Five Well-Being Index. ***P < 0.001.

Figure 3 shows the comparisons of the ESS, SNRS-11, and WHO-5 scores between the time points after BS, RS1, and RS2. The ESS score after SD (18.35 ± 0.56) was significantly higher than that after BS (5.75 ± 0.45) and after RS1 (6.6 ± 0.62) (P < 0.001 and P < 0.001, respectively) (Figure 3A). The SNRS-11 score after SD (7.65 ± 0.39) was significantly higher than that after BS (1.35 ± 0.26) and after RS1 (2.65 ± 0.40) (P < 0.001, P < 0.001) (Figure 3B). Finally, the WHO-5 score after SD (5.55 ± 0.71) was significantly lower than that after BS (19.6 ± 0.59) and after RS1 (19.15 ± 0.58) (P < 0.001 for both comparisons) (Figure 3C).

**Figure 4:**
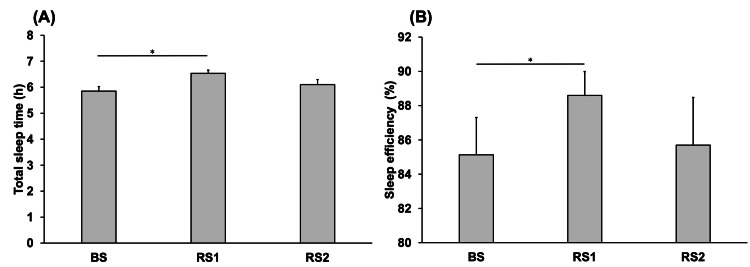
Comparison of the total sleep time (A) and sleep efficiency (B) on each day. BS: baseline sleep; RS1: recovery sleep 1; RS2: recovery sleep 2. *P < 0.05.

Figure 4 shows the comparisons of the total sleep time and sleep efficiency between the BS, RS1, and RS2 periods. The total sleep time during RS1 (6.53 ± 0.12 hours) was significantly higher than that during BS (5.85 ± 0.17 hours) (P = 0.036) (Figure 4A). The sleep efficiency during RS1 (88.6 ± 1.39) was significantly higher than that during BS (85.1 ± 2.17) (P = 0.047) (Figure 4B).

**Figure 5:**
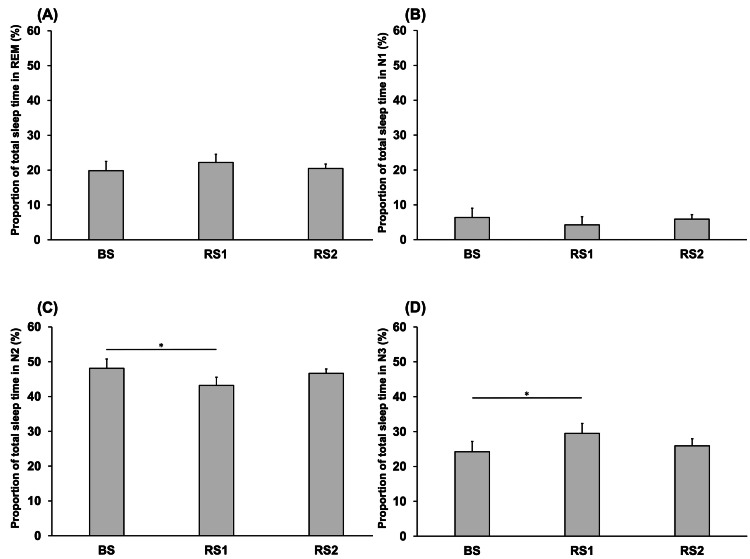
Comparison of the proportion of time spent in REM (A), proportion of time spent in N1 (B), proportion of time spent in N2 (C), and proportion of time spent in N3 (D) on each day. BS: baseline sleep; RS1: recovery sleep 1; RS2: recovery sleep 2; REM: rapid eye movement. *P < 0.05.

Figure 5 shows the comparisons of the proportions of sleep time spent in REM, N1, N2, and N3 between the BS, RS1, and RS2 periods. The proportion of sleep time spent in REM showed no significant difference between the BS (19.83 ± 1.68), RS1 (22.21 ± 1.45), and RS2 (20.47 ± 1.73) periods (P > 0.05) (Figure 5A). The proportion of sleep time spent in N1 showed no significant difference between the BS (6.37 ± 0.82), RS1 (4.26 ± 0.35), and RS2 (5.92 ± 0.63) periods (P > 0.05) (Figure 5B). The proportion of sleep time spent in the N2 during RS1 (43.1 ± 2.36) was significantly lower than that during BS (48.1 ± 2.67) (P = 0.032) (Figure 5C). Finally, the proportion of sleep time spent in N3 during RS1 (29.4 ± 2.87) was significantly higher than that during BS (24.2 ± 2.96) (P = 0.019) (Figure 5D).

## Discussion

This study investigated the effect of SD on masticatory muscle activity during sleep objectively using a portable EMG device. The key findings were as follows: (1) the number of SB events per hour and the microarousal index during RS1 were significantly lower than those measured during BS and RS2; (2) the ESS, SNRS-11, and WHO-5 scores measured after SD were significantly different from those measured after BS and after RS1; (3) the total sleep time and sleep efficiency during RS1 were significantly higher than those measured during BS; and (4) the proportions of sleep time spent in the N2 and N3 stages of non-REM sleep during RS1 were significantly different than those quantified during BS.

Previous studies have demonstrated associations between sleep quality and anxiety or depression based on cross-sectional survey data [21,22]. Kinnunen et al. [23] have suggested that sleep quality affects vigor, potentially leading to reduced stress levels. Moreover, one study has demonstrated that psychosocial factors may be risk factors for SB [24]. The results of the present study suggested that the participants’ stress levels were significantly decreased after RS1 compared to those measured after SD based on the SNRS-11 and WHO-5 scores, and the number of SB events during RS1 was significantly lower than the frequencies recorded during BS and RS2. The marked reduction in SB events observed during RS1 may be related to the rebound increase in deep sleep (N3) after SD, which temporarily improved sleep architecture. This compensatory effect appeared to diminish by RS2, during which the distribution of sleep stages returned closer to the BS level, and the number of SB events increased again. Additionally, the total sleep time and sleep efficiency during RS1 were significantly higher than those assessed during BS. Collectively, these results suggest that improving sleep quality has the potential to reduce the frequency of SB events, potentially through the alleviation of stress. However, sleep disorders are classified as chronic conditions, and some previous studies have reported that neither total sleep time nor sleep efficiency affects SB events [25,26]. The reason for these discrepancies remains unknown, and further studies are required to objectively assess the long-term impact of sleep quality on the occurrence of SB events, particularly in relation to sleep architecture and stress.

Polysomnographic studies have shown that SB events occur most often during the N2 stage of sleep, with phasic SB events being more frequent than tonic or mixed events across all stages [3]. Huynh et al. [27] suggested that the occurrence of SB events associated with arousal activity during sleep periods and the increased number of SB events observed during the ascending phase of the sleep cycle are the result of the heightened responsiveness of oromotor activity to microarousal. Recent studies have suggested that rhythmic masticatory muscle activity is not simply an abnormal event that disrupts sleep, but rather a component of the arousal-related response [28]. The present results suggest that both the number of SB events and the microarousal index during RS1 were significantly lower than those measured during the BS and RS2 periods, indicating that the onset of SB may be strongly associated with microarousals.

In this study, the proportion of time spent in the N3 stage during RS1 was significantly higher than that during BS, whereas the proportion of time spent in the N2 stage during RS1 was significantly lower than that during BS, suggesting that not only improvements in sleep quality but also alterations in the distribution of time spent in various sleep stages may affect the likelihood of SB events. SD was used to alter sleep conditions; however, further studies are needed to investigate the effect of sleep conditions on SB events to elucidate the mechanism underlying SB and to establish novel strategies for its management.

Despite the positive findings, this study has some limitations. First, the small number of participants might have limited the statistical robustness of the findings. Second, the manner in which SD was experimentally induced may not fully reflect natural sleep disturbances or stress levels. Third, this study did not classify participants based on the number of SB events or polysomnographic diagnostic cut-off criteria for SB [29]. Therefore, the sample may have included participants with severe SB or patho-bruxism [30], which could have affected the results. Further studies are needed to distinguish between normo- and patho-bruxism, to synchronize measurements of masticatory muscle activity, and to evaluate sleep architecture over a long period of time. Such studies would help clarify the relationship between sleep architecture and SB events and contribute to the development of novel interventions for SB.

## Conclusions

Ultimately, the results of this study suggest that SD can alter sleep architecture and stress status, thereby influencing the occurrence of SB events, and that improving sleep quality can reduce their frequency by alleviating stress. These findings will be useful clinically to improve the management of SB from lifestyle (i.e., the modification of sleep schedules).
